# Authors’ Response to Peer Reviews of “Improving Tuberculosis Detection in Chest X-Ray Images Through Transfer Learning and Deep Learning: Comparative Study of Convolutional Neural Network Architectures”

**DOI:** 10.2196/77221

**Published:** 2025-07-01

**Authors:** Alex Mirugwe, Lillian Tamale, Juwa Nyirenda

**Affiliations:** 1 School of Public Health, Makerere University, Kawalya Kaggwa Close, Plot 20A, Kampala, Uganda, 256 701120534; 2 Faculty of Science and Technology, Victoria University, Kampala, Uganda; 3 Department of Statistical Sciences, University of Cape Town, Cape Town, South Africa

**Keywords:** tuberculosis detection, tuberculosis, TB, chest x-ray classification, diagnostic imaging, radiology, medical imaging, convolutional neural networks, data augmentation, deep learning, early warning, early detection, comparative study

*This is the authors’ response to peer-review reports for “Improving Tuberculosis Detection in Chest X-Ray Images Through Transfer Learning and Deep Learning: Comparative Study of Convolutional Neural Network Architectures.”*

## Round 1 Review

### Reviewer AE [1]

#### General Comments

##### Clarity and Structure

*The paper [2] presents a comprehensive overview of the methods and results but can benefit from clearer transitions between sections. For instance, adding brief connecting sentences at the end of each section would help guide the reader into the next topic.*

*Consider reorganizing the “Discussion” section to first summarize the key findings before delving into their implications. This will reinforce the reader’s understanding of the main outcomes.*

##### Writing Style

*Aim for more active voice usage to enhance readability. For example, change “It was observed that VGG16 outperformed other models” to “We observed that VGG16 outperformed other models.”*

*Simplify overly technical or long sentences to improve readability. Breaking complex sentences into two simpler ones can make the content easier to follow.*

**Response:** We have revised the manuscript to improve transitions between sections by adding concluding statements that summarize key points and guide the reader to the next section. Regarding the Discussion section, we believe the current structure effectively presents the findings and their implications. The key outcomes are already summarized at the start of the section, followed by a detailed discussion of their clinical and technical implications.

### Specific Comments by Section

#### Abstract

*Sentence clarification: The phrase “necessitating more efficient and accurate diagnostic methods” could be expanded to briefly indicate why current methods are insufficient.*

*Results detail: When mentioning model performance, briefly state why VGG16’s superior performance is significant compared to others.*

**Response:** We have revised the abstract to enhance its clarity and readability. Additionally, we included a clear Objective section to directly address the comment and make the study’s purpose more explicit.

For sentence clarification, we have revised the Introduction section of the abstract to clearly indicate why current diagnostic methods are insufficient. For results details, we have revised the Results section of the abstract to explain why VGG16’s superior performance was significant, emphasizing its balance of diagnostic accuracy and computational efficiency.

#### Introduction

*Background information: The explanation of the global tuberculosis (TB) burden is informative, but it could benefit from briefly mentioning current limitations in artificial intelligence–based TB detection in developing countries.Motivation clarification: Ensure that the motivation for choosing specific convolutional neural network architectures is clearly linked to gaps in existing literature.*

**Response:** We have revised the Introduction section to expand on the paragraphs, addressing the limitations of artificial intelligence–based TB detection in developing countries and clarifying the motivation for choosing specific convolutional neural network (CNN) architectures.

#### Methods

*Preprocessing details: The detailed explanation of normalization and data augmentation is excellent, but it might be beneficial to briefly mention how these choices align with previous research findings or unique aspects of this study.*

*Transfer learning: Include a brief comparison of why transfer learning was chosen over training models from scratch.*

**Response:** We have revised the Pre-Processing section to incorporate findings from previous research in the Normalization and Data Augmentation subsections, emphasizing how these techniques address unique aspects of this study, such as dataset imbalance and real-world variability in chest x-ray data. For the Transfer Learning section, we added a brief comparison explaining why transfer learning was preferred over training models from scratch, highlighting its advantages in resource-limited settings and its proven effectiveness in medical imaging tasks.

#### Results

*Visualization: The table summarizing model performance is comprehensive, but consider including a concise narrative to describe key trends observed in the data.*

*Analysis clarification: When discussing why data augmentation did not enhance performance, elaborate on how this aligns with or contradicts findings from other studies.*

#### Discussion

*Comparison with previous studies: Add a few sentences comparing the results with existing studies that used the same models or datasets to provide context.*

*Implications: Discuss the practical implications of using VGG16 in resource-constrained environments where computational efficiency is crucial.*

#### Conclusion

*Highlight novelty: Emphasize what makes this study’s approach unique, such as the use of specific architectures on a larger dataset, and how this adds to the current body of knowledge.*

*Future work suggestions: Include more detailed recommendations for future studies, potentially suggesting how to further leverage data augmentation strategies.*

**Response:** We have revised the Discussion section to include two additional paragraphs elaborating on why data augmentation did not improve performance. These paragraphs provide a detailed explanation of how our findings align with certain previous studies while contrasting with others.

### Reviewer AI [3]

*1. The dataset includes a large imbalance between TB-positive and TB-negative images (700 vs 3500). Explain how this imbalance was addressed beyond augmentation or whether balancing techniques like oversampling were considered.*

**Response:** No additional balancing methods were used, such as oversampling or undersampling. Instead, data augmentation was specifically used to introduce variability and enhance the representation of TB-positive images, constituting the smaller class. Given the study’s objectives and dataset characteristics, this approach was considered adequate for addressing the class imbalance.

*2. While each architecture’s parameters are listed, there is no in-depth discussion on why these specific parameters (eg, dropout rates, learning rates) were selected.*

**Response:** A paragraph has been added at the end of the CNN Architectures subsection to explain how we arrived at the parameters used for training. This addition clarifies that the parameters were determined through a rigorous iterative process of experimentation and were selected based on their ability to deliver optimal performance across the evaluated architectures.

*3. The conclusion that data augmentation did not improve performance lacks specific references to possible reasons.*

**Response:** We have added a detailed explanation in the Discussion section, citing studies that achieved similar results and those with augmentation improved performance. We have also explained why the latter was not the case in our study.

*4. While computational time for each model is reported, further analysis of the practical implications, such as cost-effectiveness for clinical settings, is missing.*

**Response:** In response to the comment regarding the practical implications of computational time, we have added a paragraph in the Discussion section to address cost-effectiveness and the relevance of model training times for clinical settings.

*5. The manuscript mentions transfer learning with pretrained ImageNet weights, but there is limited information on why this was the chosen approach versus training from scratch.*

**Response:** We added a brief comparison explaining why transfer learning was preferred over training models from scratch, highlighting its advantages in resource-limited settings and its proven effectiveness in medical imaging tasks.

*6. Throughout the Results section, adding comparative charts or visual aids for each model’s performance across metrics like accuracy, precision, and area under the receiver operating characteristic curve would improve readability.*

*7. The Conclusion could benefit from a clearer statement on how these findings advance the field of TB detection in medical imaging.*

**Response:** Your suggestions have been addressed by adding more clarity to the Results, Discussion, and Conclusion sections.

## Abbreviations

CNN: convolutional neural network
TB: tuberculosis
